# GOgetter: A pipeline for summarizing and visualizing GO slim annotations for plant genetic data

**DOI:** 10.1002/aps3.11536

**Published:** 2023-08-11

**Authors:** Emily B. Sessa, Rishi R. Masalia, Nils Arrigo, Michael S. Barker, Jessie A. Pelosi

**Affiliations:** ^1^ New York Botanical Garden Bronx New York USA; ^2^ Department of Ecology and Evolutionary Biology University of Arizona Tucson Arizona USA; ^3^ SOPHiA Genetics Saint Sulpice Switzerland; ^4^ Department of Biology University of Florida Gainesville Florida USA

**Keywords:** annotation, data mining, gene function, gene ontology

## Abstract

**Premise:**

The functional annotation of genes is a crucial component of genomic analyses. A common way to summarize functional annotations is with hierarchical gene ontologies, such as the Gene Ontology (GO) Resource. GO includes information about the cellular location, molecular function(s), and products/processes that genes produce or are involved in. For a set of genes, summarizing GO annotations using pre‐defined, higher‐order terms (GO slims) is often desirable in order to characterize the overall function of the data set, and it is impractical to do this manually.

**Methods and Results:**

The GOgetter pipeline consists of bash and Python scripts. From an input FASTA file of nucleotide gene sequences, it outputs text and image files that list (1) the best hit for each input gene in a set of reference gene models, (2) all GO terms and annotations associated with those hits, and (3) a summary and visualization of GO slim categories for the data set. These output files can be queried further and analyzed statistically, depending on the downstream need(s).

**Conclusions:**

GO annotations are a widely used “universal language” for describing gene functions and products. GOgetter is a fast and easy‐to‐implement pipeline for obtaining, summarizing, and visualizing GO slim categories associated with a set of genes.

The functional annotation of genetic data is a critical component of ‐omics research, but the primary process of assigning functional annotations (henceforth “annotation”) to a gene or set of genes in a genome is labor‐ and time‐intensive, expensive, and requires extensive experimental work to determine genes’ functions or products, and the processes in which they are involved. As a result, relatively few species (e.g., *Arabidopsis thaliana*, *Drosophila melanogaster*) have genomes annotated at this level (de Crécy‐Lagard et al., 2022), and these are typically used as references from which annotation information is transferred to other data sets of interest based on the results of a sequence similarity/homology search. The result of this transfer process is typically an exhaustive list of annotations for the input sequence set; depending on the goals of subsequent analyses, these may be used in their raw form, or it may be useful or necessary to summarize and organize those raw data in some way.

Structured, hierarchical vocabularies, or ontologies, are widely used in the biological sciences for organizing and classifying terminology about biological systems (Howard et al., 2021). Ontologies consist of terms and the connections between them, which define their relationships; these structures are hierarchical in the sense that parent–child relationships exist, with broader categories serving as parents for more specialized child terms. One of the most widely used biological ontologies is the Gene Ontology (Harris et al., 2004), which includes information about three aspects of genes and gene products: their molecular function, cellular location, and the larger biological process(es) that they participate in or to which they contribute. A given gene or gene product typically has multiple annotations in the Gene Ontology (GO) that describe these various aspects and that collectively provide a wealth of information about the gene and its products’ structure, function, and location. Individual GO terms can be grouped into higher‐level sets that summarize and organize annotations according to broad categories, called GO slim categories or GO slims. While an individual gene's annotations may fall into multiple GO slims, the categories themselves are non‐overlapping and collectively give a broad overview of the functions, locations, and biological processes of a data set's genes and gene products.

Given the value of GO for describing gene products and functions, obtaining the complete list of GO annotations and a summary of GO slims for a set of sequence data is highly desirable. Various lists of GO annotations are available online, including complete lists of all GO terms (e.g., from QuickGO, https://www.ebi.ac.uk/QuickGO/annotations [Binns et al., 2009]) and numerous taxon‐specific lists (available from the Gene Ontology Consortium, http://current.geneontology.org/products/pages/downloads.html [Ashburner et al., 2000; Gene Ontology Consortium, 2023], and other sources). However, downloading a reference list of annotations is only the first step; a user needs to query their own data against the reference set to determine the annotations, and then identify the GO slim categories of those annotations. There are several existing tools and software suites that can be used to obtain annotations for a set of genes. Many of these packages first require the use of a tool for sequence similarity/homology searches (such as BLAST; Altschul et al., 1990), and will then assign GO terms to a set of known coding sequences. Packages with this capability include Blast2GO (a platform which requires a paid subscription; Conesa et al., 2005) and agriGO (which was developed primarily for agricultural plants; Tian et al., 2017). Trinotate (Bryant et al., 2017) is perhaps the most widely used functional annotation software for transcriptomes and integrates a variety of tools (e.g., BLAST, HMMER [Eddy, 2011], signalP [Petersen et al., 2011], and tmHMM [Krogh et al., 2001]) to associate putative genes to functions based on database searches. These programs, however, leave the user with a list of raw GO terms for their set of input data. While such data certainly have use, the characterization of gene sets to GO slims can be vital for visualizing data and answering biological questions (see, for example: Barker et al., 2008; Li et al., 2018; Marx et al., 2021; Pelosi et al., 2022).

Various web‐based tools are available to characterize a set of genes from a model organism (GOTermMapper; https://go.princeton.edu/cgi-bin/GOTermMapper), to summarize GO terms to GO slims based on a pre‐defined GO slim set (GOSlimViewer; https://agbase.arizona.edu/help/slimviewerhelp.htm), or to calculate data set–specific summaries (REVIGO; Supek et al., 2011). However, a command‐line tool that combines all of the above aspects (i.e., sequence similarity searching through to characterization of GO slims) is lacking. Here we introduce GOgetter, a set of Python and bash scripts that uses BLAST for sequence homology searches of an input FASTA file of nucleotide gene sequences and characterizes the functional categories of the input at the level of GO slims with both text and visualization files as output. GOgetter allows the user to use any database and set of GO terms to characterize their data, giving it an advantage over other existing software that predefine these. GOgetter is a freely available, easy‐to‐use, and flexible tool for the functional annotation of plant genetic data to GO slims.

## METHODS AND RESULTS

### Computational requirements and implementation of GOgetter

GOgetter was designed to provide the most straightforward path from a FASTA file of nucleotide coding sequence data to an output text file summarizing the GO slim annotations of the input. The pipeline was written in Python v3.8, is open source, and is available at https://github.com/jessiepelosi/GOGetter under a GNU Public License. GOgetter requires the Python packages argparse v1.1, matplotlib v3.3.0, numpy v1.20.2, pandas v1.2.4, re v2.2.1, and seaborn v0.11.2, and was tested on the University of Florida's HiPerGator computing cluster with Linux on an AMD EPYC 75F3 Milan with 3.0 GHz cores, 8 GB/core, and using BLAST v2.10.1 for sequence similarity/homology searches. Any operating system capable of running Python and BLAST should be capable of supporting GOgetter. A user manual (README.md) and directory containing example files are included in the GitHub repository. The example directory includes example input FASTA files, which are subsets of coding sequences for three publicly available fern species (*Dryopteris decipiens* (Hook.) Kuntze, FASTA filename prefix DRDE; *Lygodium japonicum* (Thunb.) Sw., LYJA; and *Vittaria appalachiana* Farrar & Mickel, NDUV) that have been published in previous studies (Qi et al., 2018; One Thousand Plant Transcriptomes Initiative, 2019) and outputs generated from those files using GOgetter.

**Figure 1:**
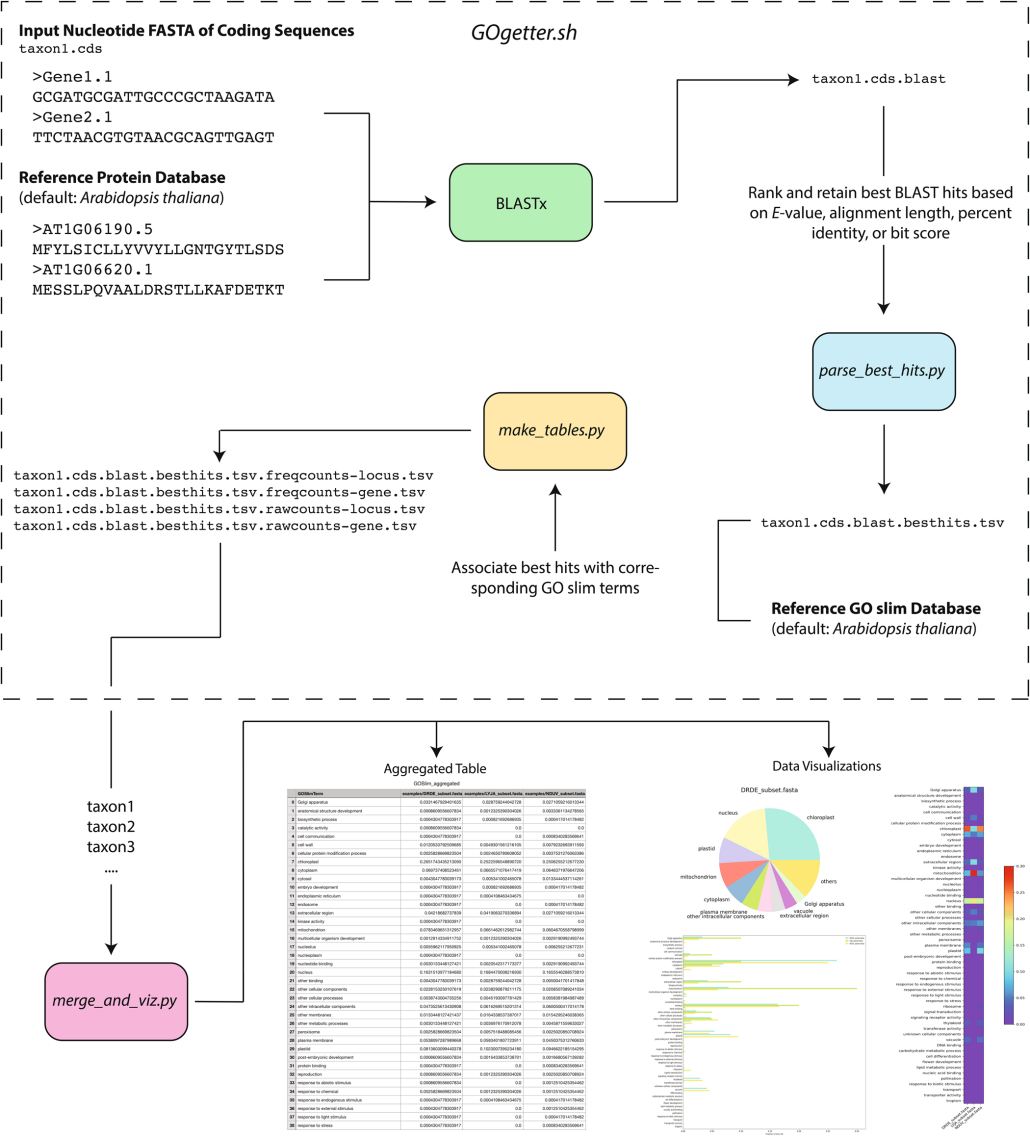
The GOgetter pipeline. Inputs, primary pipeline activity, and output file names (.tsv tables for raw and frequency count data) from *GOgetter.sh* are shown inside the dotted‐line box. The bottom of the figure shows the merged table and example figures produced from several frequency count tables using *merge_and_viz.py*, after multiple input files have been run through the pipeline.

Once the pipeline is downloaded, it consists locally of a primary GOgetter directory that contains the various pipeline scripts, as well as the subdirectory TAIR_2021 that contains a default reference database of *Arabidopsis thaliana* (L.) Heynh. gene models from The Arabidopsis Information Resource (TAIR; https://www.arabidopsis.org/ [accessed 13 February 2023]). For each input file, GOgetter can be run from the command line with *GOgetter.sh*, which (1) uses BLAST (or DIAMOND) to find significant sequence similarity/homology matches, (2) parses the best BLAST hit for each sequence (*parse_best_hits.py*), and (3) generates summary tables that characterize the GO slim composition of the input (*make _tables.py*; Figure 1). An additional Python script (*merge_and_viz.py*) can be run to merge and visualize the resulting summary information from multiple input files (Figure 1).

### Sequence similarity/homology searching

GOgetter requires a single file of genes/coding loci in a nucleotide FASTA file as input (provided by the user; Figure 1, “Input Nucleotide FASTA of Coding Sequences”), a protein BLAST database (Figure 1, “Reference Protein Database”), and corresponding GO slim mapping file (Figure 1, “Reference GO slim Database”). The BLAST database and mapping files are provided for *Arabidopsis thaliana* by default, and users can generate their own database and mapping files if desired (see Appendix 1). The first step begins with a BLASTx search of the query (“Input Nucleotide FASTA of Coding Sequences”) against the subject (“Reference Protein Database”) with a set of default parameters (‐num_threads 1 ‐max_target_seqs 50 ‐evalue 0.001) that can be changed by the user when executing *GOgetter.sh*. This is a relatively lenient set of metrics for the BLAST search; more stringent filters can be applied when determining the best BLAST hit for each input gene sequence in *parse_best_hits.py*. An alternative sequence similarity search program, such as DIAMOND (Buchfink et al., 2021), may be run in place of BLAST and called from *GOgetter.sh* by using the ‘‐n’ flag. While DIAMOND is a faster alternative to BLAST, it is less accurate (Buchfink et al., 2015) and therefore BLAST is used as the default search program by GOgetter (see Case Study 1 for the differences in BLAST vs. DIAMOND searches). Those wishing to use additional parameters may run these searches independent of *GOgetter.sh*, as long as the output format of the searches matches ‐outfmt 6 of BLAST.

### Parsing best hits and summarizing GO slim composition

The output BLAST search is then, by default, filtered to remove matches with *E*‐values greater than 1 × 10^−5^. By including this additional filtering step in *parse_best_hits.py*, the user can run the first BLAST step once (which is the most memory‐ and time‐consuming step), and can then adjust the filtering parameters in *parse_best_hits.py* to generate multiple different outputs based on varying *E*‐value cutoffs, rather than rerunning the whole BLAST search. By default, *parse_best_hits.py* filters BLAST hits based on *E*‐value alone, which is the most common and frequently used metric for assessing significance of a similarity search. However, additional metrics can be used to filter, including the alignment length, the percent identity, and bitscore; the default values of these metrics are set to 0 but can be changed from the command line. We use these defaults as it is not possible to develop a universal set of cut‐offs given that users may be interested in different gene families with variable protein lengths and/or use inputs with varying divergence from the reference.

The resulting matches are sorted and ranked, and the best hit data are written to a tab‐delimited output file containing the query and subject. Ranking of BLAST hits can be based on the lowest *E*‐value (default), highest bit score, longest alignment length, or highest percent identity. We used the *E*‐value as the default ranking parameter because it is a commonly used metric to assess the significance of BLAST hits and is corrected for database size and query length, although other metrics may be preferred (Pearson, 2013). The script *make_tables.py* is next used to summarize the number and frequency of each GO slim category present in the input gene set. This portion of the pipeline requires a reference mapping file from each gene/locus in the BLAST database to the corresponding GO slim category (“Reference GO slim Database”). The output of *make_tables.py* consists of four table files: raw and frequency count tables that include all GO slim categories, at both gene‐ and locus‐levels as defined by TAIR.

### Merging summary tables and visualizing results

**Figure 2:**
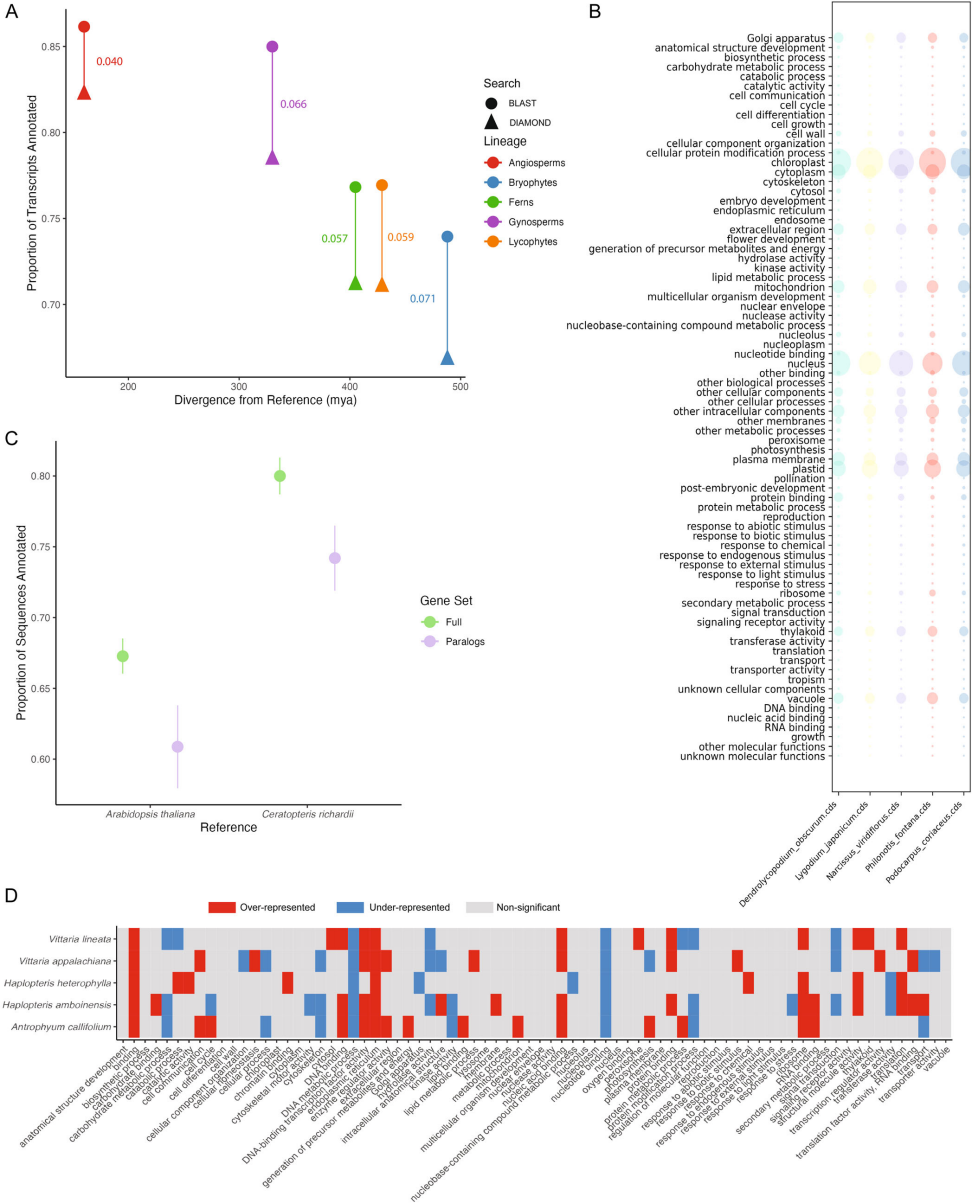
Results of case studies using GOgetter: (A, B) Case Study 1, (C, D) Case Study 3. (A) Proportion of transcripts annotated to a GO slim term using GOgetter with BLAST (circle) and DIAMOND (triangle) for five transcriptomes representing angiosperms (red), gymnosperms (purple), ferns (green), lycophytes (orange), and bryophytes (blue). Vertical lines connecting points show the difference in the proportion of sequences annotated between BLAST and DIAMOND searches. (B) Bubble graph generated from GOgetter for the five transcriptomes analyzed, scaled to the proportion of total annotations that associate with that term. (C) The average proportion of sequences annotated to a GO slim term using GOgetter for full transcriptomes (green) and paralogs (purple) for five species of vittarioid ferns, using either *Arabidopsis thaliana* (left) or *Ceratopteris richardii* (right) as a reference. Error bars show ±1 standard error. (D) Heatmap of significantly over‐ (red) and under‐represented (blue) GO slim categories in paralog sequences relative to full transcriptomes. Non‐significant differences between the paralog set compared to the full transcriptome are depicted as gray.

*GOgetter.sh* calls the BLAST command and the two Python scripts described above for one set of genes. Users wanting to compare multiple gene sets can run the pipeline as a for‐loop or an array (as on a computing cluster). The additional Python script *merge_and_viz.py* can then be used to perform an outer merge over a set of output tables produced by *GOgetter.sh* from multiple individual inputs. The output of this step consists of a tab‐delimited file with the first column composed of the GO slim categories followed by columns with corresponding frequency or count data for each input gene set. By using the “both” runmode (‐m both), users will generate this table and several visualizations of the data including pie charts, bar plots, heatmaps (Figure 1), and bubble graphs (Figure 2B). Given that plastid transcripts are very abundant (up to 82% of the cellular mRNA pool; Forsythe et al., 2022) and may swamp out contributions from other functional categories, both raw and log‐transformed heatmaps are generated. The merged output data format allows for easy input into other programs for downstream statistical analysis that are not included in GOgetter.

### Case studies

We performed three case studies to demonstrate the behavior and usage of GOgetter under several different circumstances and parameters, and to illustrate how GOgetter may be applied to answer biological questions. In Case Study 1, we show that GOgetter is widely applicable across land plants and explore the differences observed when using BLAST and DIAMOND search algorithms, as well as the effect of differing search programs on the resulting characterization of gene sets to higher‐order GO slims. In Case Study 2, we examine the effects of varying the stringency of the filtering parameters on GOgetter results. In Case Study 3, we illustrate how GOgetter can be used to explore biological systems by investigating differential functions of genes retained in duplicate following putative whole genome duplication events and the suitability of the reference database employed.

#### Case Study 1: Applicability across land plants

We downloaded transcriptomes from the One Thousand Plant Transcriptomes Initiative (2019) and Qi et al. (2018) for representative non‐model species for each of the major lineages of land plants: *Philonotis fontana* (Hedw.) Brid. (bryophytes), *Dendrolycopodium obscurum* (L.) A. Haines (lycophytes), *Lygodium japonicum* (ferns), *Podocarpus coriaceus* Rich. & A. Rich. (gymnosperms), and *Narcissus viridiflorus* Schousb. (angiosperms). We used TransDecoder v5.50 (https://github.com/TransDecoder/TransDecoder) to extract coding sequences (CDS) from the 1KP assemblies; data from Qi et al. (2018) were already available as coding sequences. We ran GOgetter on each CDS with the default parameters, once with BLAST v2.10.1 (Altschul et al., 1990) and once with DIAMOND v2.1.3 (Buchfink et al., 2021). To investigate both the potential impact of divergence level between the subject and reference used in GOgetter, and the divergence times between the study species and *Arabidopsis thaliana* (the default reference in GOgetter), we used the median ages from TimeTree5 (Kumar et al., 2022).

GOgetter was successful in assigning GO slims to 73.86% to 86.02% of transcripts when run with BLAST and 66.75% to 82.18% of transcripts when using DIAMOND for these five species. On average, DIAMOND recovered 62.42% fewer hits and 5.86% fewer transcripts were annotated compared to the BLAST searches (Figure 2A). Unsurprisingly, the divergence between the reference (*Arabidopsis thaliana*) and the subject had a significant impact on the proportion of transcripts annotated (adjusted *R* ^2^ = 0.624, *F*_1,8_ = 15.95, *P* = 0.004; Figure 2A) (see Case Study 3 for considerations of other reference databases). Despite these differences, we found that there were no significant differences in the proportion of GO slims in each of the study systems when using BLAST or DIAMOND (χ^2^ = 40.55–56.70, *df* = 72, *P* > 0.9; Figure 2B, results using BLAST shown). This case study demonstrates the wide applicability of GOgetter to categorize the functions of a gene set across land plants diverged over 480 mya when the default *Arabidopsis thaliana* is used as the reference, and shows that the categorization of GO slims is relatively robust to different sensitivities of the similarity/homology search algorithms used.

#### Case Study 2: Parameters in similarity searches and resulting higher‐order functional categories

We further examined how changing the filtering parameters impacts higher‐order GO slim categories summarized by GOgetter. We ran *GOgetter.sh* using the *Lygodium japonicum* CDS with default parameters to get a resulting set of BLAST hits. We then determined the best hits based on several different filtering criteria by running *parse_best_hits.py* and *make_tables.py* to generate new summary tables for each set of filtering criteria. We explored the effects of the individual metrics by filtering using: *E*‐value (cutoffs: 1 × 10^−5^, 1 × 10^−10^, 1 × 10^−15^, 1 × 10^−20^), alignment length (30, 50, 100, 150), percent identity (10, 25, 33, 40), and bitscore (33, 50, 60, 75). We then combined metrics to generate four sets of filtering criteria from least stringent (*E*‐value < 1 × 10^−5^, alignment length > 30, percent identity > 10, bitscore > 33) to most stringent (*E*‐value < 1 × 10^−5^, alignment length > 150, percent identity > 40, bitscore > 75). We used *merge_and_viz.py* to merge the tables generated from each assessment (e.g., all *E*‐value tables were merged or all bitscore tables were merged) and used χ^2^ tests to compare the results and determine whether there was a significant impact of filtering BLAST hits using different stringencies on the resulting annotation to GO slim terms. Although there were substantial changes in the proportions of BLAST hits passing these filtering cutoffs and the number of transcripts annotated (Appendix S1), there were no significant differences in the annotation to GO slim terms as the metric cutoffs changed for *E*‐value, alignment length, percent identity, or bitscore (all *P* > 0.05).

When we combined different metrics for filtering (i.e., filtering based on a combination of *E*‐value, alignment length, percent identity, and bitscore cutoffs), we observed a more drastic effect on the number of BLAST hits passing the cutoffs (least stringent: 89.91% passing; most stringent: 10.90% passing) and the number of transcripts annotated (least stringent: 76.66% annotated; most stringent: 43.73% annotated). There was a significant difference in the proportions of sequences annotated to GO slim terms (χ^2^ = 261.4, *df* = 210, *P* = 0.009), but only when including the most stringent filtering; otherwise, there were no significant differences (χ^2^ = 49.588, *df* = 140, *P* = 1.0) when comparing only the three less stringent filter combinations. The differences we observed due to differences in filtering may arise due to the fraction of the CDS that is annotated; in the three less stringent filters, between 64.3% and 76.7% of the transcripts were annotated to a GO slim category, whereas only 43.7% transcripts were annotated in the most stringent filter. This second case study demonstrates that the categorization of GO slims in a gene set is relatively robust to changes in the filtering parameters employed, although more stringent filtering may result in more pronounced effects on the functional categorization of the gene set.

#### Case Study 3: Selecting a suitable reference database

For five species of vittarioid ferns, we retrieved whole transcriptomes and sets of duplicate genes (putative paralogs) retained following a vittarioid‐specific whole genome duplication from Pelosi et al. (2022). To explore the effects of using different reference databases in the BLAST step, we ran the GOgetter pipeline for each input file using two reference species: *Arabidopsis thaliana* and *Ceratopteris richardii* Brongn. We chose to use the *Ceratopteris* genome as this species is a model fern (Kinosian and Wolf, 2022), has a well‐annotated genome, and is more closely related to the vittarioid ferns than is *Arabidopsis*, with a divergence time of 105 mya vs. 405 mya. For *Ceratopteris*, we followed the steps described in Appendix 1 to generate a custom GO slim database using *Ceratopteris richardii* v2.1 (Marchant et al., 2022) for the BLAST database (files obtained from https://phytozome-next.jgi.doe.gov/info/Crichardii_v2_1). The ‘plant_goslim’ list from QuickGO (Binns et al., 2009) was used for the GO slim mapping database (see Appendix 1), excluding terms related to flowers and fruits: GO:0009908, flower development; GO:0009856, pollination; and GO:0009835, fruit ripening. Each gene set was passed through GOgetter with BLAST as the similarity/homology search program, and hits were filtered to remove those with *E*‐values > 1 × 10^−5^ and alignment lengths < 100.

We found that there was a significantly lower proportion of sequences annotated (*F*_1,16_ = 39.642, *P* = 1.06 × 10^−5^) in the paralog gene sets compared to the full transcriptomes (*F*_1,16_ = 8.712, *P* = 0.00938) when we used *Arabidopsis* as the reference, but there was no interaction between the reference used and the gene set (*F*_1,16_ = 0.021, *P* = 0.88695) (Figure 2C). Interestingly, we did not see a significant impact of the reference database on the proportion of hits passing (*F*_1,16_ = 1.206, *P* = 0.2886), but the proportion of hits passing was significantly less for the paralogs compared to full transcriptomes (*F*_1,16_ = 6.084, *P* = 0.0253). The differences observed between the full transcriptomes and paralog sets may be a result of fragmentation or mutation in paralogs over time following whole genome duplication and subsequent failure to assign an annotation because of these factors.

We compared the composition of each pair of gene sets (full transcriptome and putative paralogs) for each species individually using *Ceratopteris richardii* as the reference. In every case, there were significant differences in the overall functional categorization between full transcriptomes and paralogs (*P* < 10^−16^). Following Barker et al. (2008) and Shi et al. (2010), GO slim categories with χ^2^ residuals greater than 2 are considered to be over‐represented and those with residuals less than –2 are considered to be under‐represented in the paralog gene set relative to the full transcriptome. In general, we found that the functions of genes retained in duplicate following this whole genome duplication were largely convergent across species (Figure 2D). There were several GO slims that showed either over‐representation (e.g., binding, endoplasmic reticulum, ribosome) or under‐representation (e.g., nucleotide binding, metabolic process, protein modification process) in the paralogs of all or most species (Figure 2D). In other cases, there were opposite trends, where categories were over‐represented in one species but under‐represented in another (e.g., cell cycle, transport; Figure 2D), suggesting that there may be lineage‐specific processes that have shaped genome evolution following the vittarioid‐specific whole genome duplication (Pelosi et al., 2022).

## CONSIDERATIONS

GOgetter does not perform gene prediction, structural annotation, or any statistical analyses; rather this pipeline was developed to provide a straightforward method for generating a summary of functions for a set of input gene sequences. As we have shown, the characterization of gene sets to higher‐order functional categories (i.e., GO slims) is relatively robust to differing sensitivities in sequence similarity searches, filtering criteria, and divergence from the reference database. While the case studies show that results are stable, users should think critically before running any annotation software, including GOgetter. Importantly, sequence similarity does not translate directly to gene function; the true function of a gene product cannot be determined based on sequence similarity/homology searches alone and requires extensive experimental work to fully ascertain. Furthermore, differing selective regimes on duplicated gene copies (reviewed by Li et al., 2021) can lead to different functions in organisms diverged by millions of years. Given these caveats, functional annotation of genes is still largely based on the transfer of annotations inferred from sequence similarity/homology. We recommend that users employing GOgetter for annotation of gene sets to GO slims: (1) employ BLAST rather than DIAMOND, given the disparity in the number of significant hits and the proportion of input sequences annotated to a GO slim; (2) use an appropriate reference database; and (3) determine the effect of altering filtering criteria before using the defaults, which are lenient.

## CONCLUSIONS

GOgetter is a lightweight pipeline that provides the user with an easy‐to‐use method for obtaining, summarizing, and visualizing a complete set of GO slim category annotations for a set of input nucleotide sequence data. The sequence data can be generated by any method, and could come from any organism, although the pipeline is set by default to use annotations from *Arabidopsis thaliana* as a reference. The output table files provide a summary of GO slim category annotations in both raw count and frequency format, which can be used in downstream statistical analyses. The visualization step provides publication‐ready figures, including comparisons of the distribution of GO slim categories across input files. This pipeline is the first to provide such a tool for summarizing and illustrating GO slim annotation information directly from nucleotide sequence data.

## AUTHOR CONTRIBUTIONS

N.A., R.R.M., and M.S.B. wrote the original GOgetter scripts. E.B.S. and J.A.P. updated and finalized the pipeline. E.B.S. and J.A.P. drafted the manuscript, and all authors contributed to and approved the final version.

## Supporting information


**Appendix S1**. Changes in the proportion of BLAST hits returned (left column) and transcripts annotated (right column) for various values of four filtering criteria (top row to bottom row: *E*‐value, alignment length, bitscore, percent identity).

## Data Availability

GOgetter is available under a GNU General Public License (GPL) at https://github.com/jessiepelosi/GOGetter.

## Appendix 1 Instructions for generating the custom Reference Protein Database and Reference GO slim Database files.

1. A set of reference proteins in a single FASTA file (usually in the form of representative gene models of a genome) can be used to generate a BLAST database using the following command:
  makeblastdb ‐in [taxon.fasta.pep] ‐parse_seqids ‐title “[Taxon] Database” ‐dbtype prot
2. Two options are available for generating a custom GO slim mapping file.
  a. Users can generate a GO slim mapping file from a set of genes annotated to GO terms and a predefined set of GO slim categories; a Plant GO slim (‘plant_goslim’) is already available. From the main page of QuickGO (https://www.ebi.ac.uk/QuickGO/), select “Explore Biology” and then “Predefined GO slims”. Here, we choose “goslim_plant” as an example, and then select “Apply”. Refine the selection on the next page as needed (i.e., by removing irrelevant categories as described above, such as flowering‐related terms in an analysis of ferns) and export as a tab‐delimited file (TSV). The “GO TERM” column corresponds to the GO slim term for the associated GO term in the “SLIMMED FROM” column. A separate table with the GO slim term and the GO slim name can also be used to merge and associate the name to the term.
  b. QuickGO provides several documents on how to generate a custom GO slim database from a set of predefined GO terms (https://www.ebi.ac.uk/QuickGO/help/slims). Users can input the GO terms they wish to use as GO slim categories and filter the associated GO terms to each category by selecting “Explore Biology”, switching to “Input Your Own”, and pasting the GO terms of interest. The resulting table can be filtered based on the user's preferences and exported as a tab‐delimited file (TSV). The “GO TERM” column corresponds to the GO slim term for the associated GO term in the “SLIMMED FROM” column. A separate table with the GO slim term and the GO slim name can also be used to merge and associate the name to the term.
3. Associate the gene/locus information with the accompanying GO slim term using a merge function in R or Python. The annotated gene set should have columns for the locus, gene, and corresponding GO term (this can be renamed to “SLIMMED FROM” for the next step). Tables can be merged on the “SLIMMED FROM” columns that will associate the GO term (and therefore the gene/locus with a GO slim term). The resulting tab‐delimited file can be further edited for use as the GO slim mapping file required by GOgetter.
4. The custom GO slim mapping file must follow the format used in the TAIR *Arabidopsis thaliana* file (ATH_GO_GOSLIM_2021.txt). This file consists of 15 columns (see https://www.arabidopsis.org/download_files/GO_and_PO_Annotations/Gene_Ontology_Annotations/ATH_GO.README.txt for descriptions of each column). The gene/locus of the reference should be in the “ObjectName” column and the corresponding GO slim term should be in the “GOSlimTerm” column. While the other columns must be present, they can be left empty. The gene/locus name must follow the formatting of the *Arabidopsis thaliana* annotation (e.g., locus AT1G01010; gene AT1G01010.1). A string of letters, numbers, and underscores may be used in the locus name, and the gene should be the locus name followed by a period and a digit. Names of loci and genes should also be adjusted to follow this system in the input FASTA file used as the BLAST database.
