# Supplementary material for: GOgetter: A pipeline for summarizing and visualizing GO slim annotations for plant genetic data
Source: Appl Plant Sci. 2023 Aug 11;11(4):e11536. doi: 10.1002/aps3.11536 (PMC10439822; doi:10.1002/aps3.11536)

DOI 10.1002/aps3.11536

APPENDIX S1. Changes in the proportion of BLAST hits returned (left column) and transcripts annotated (right column) for various values of four filtering criteria (top row to bottom row: *E*-value, alignment length, bitscore, percent identity).

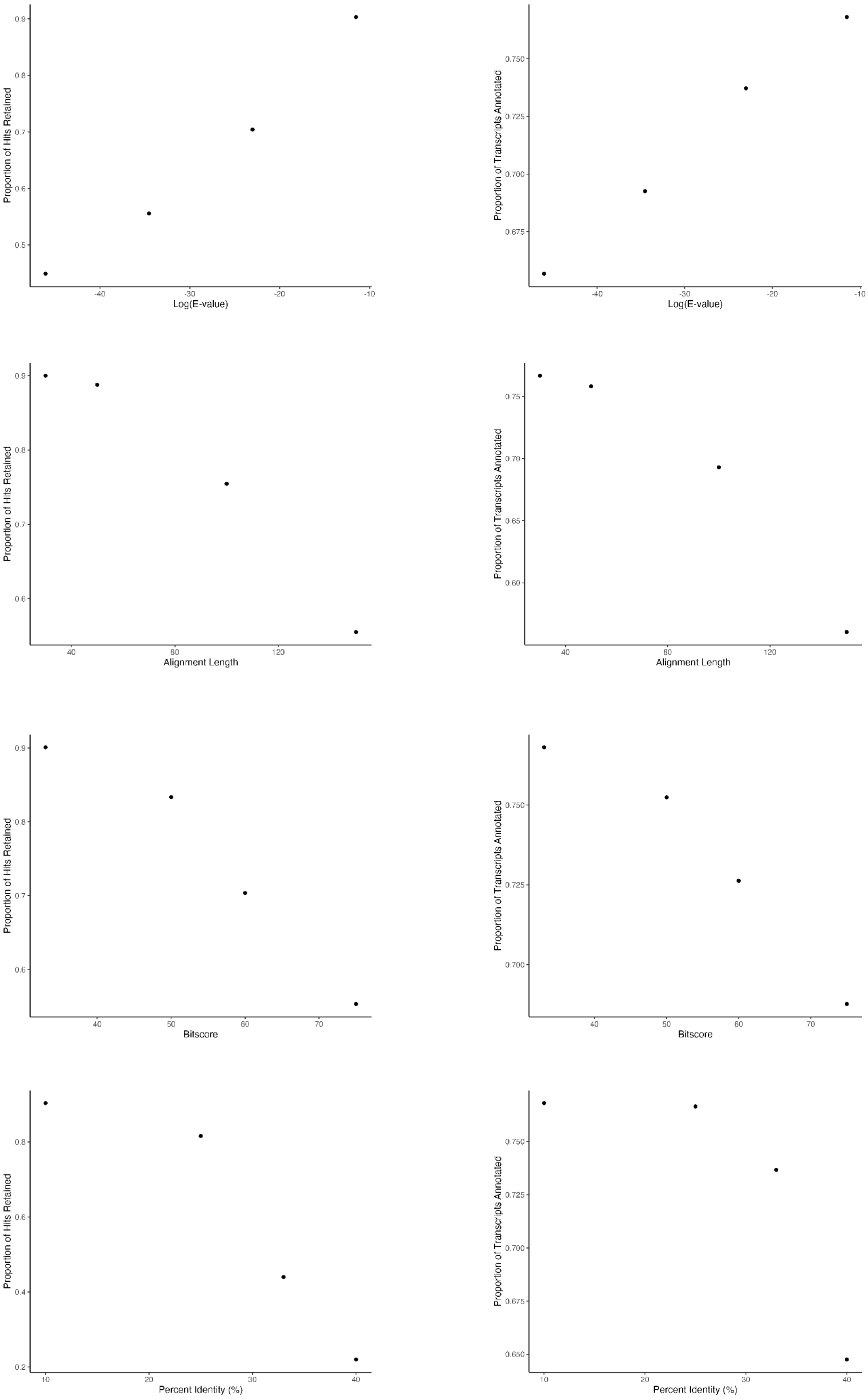

Supplement: Supplementary file 1 — Appendix S1. Changes in the proportion of BLAST hits returned (left column) and transcripts annotated (right column) for various values of four filtering criteria (top row to bottom row: E‐value, alignment length, bitscore, percent identity). [file APS3-11-e11536-s001.pdf]
